# Hepatitis C virus core protein modulates pRb2/p130 expression in human hepatocellular carcinoma cell lines through promoter methylation

**DOI:** 10.1186/s13046-015-0255-1

**Published:** 2015-11-14

**Authors:** Anna Maria Mileo, Stefano Mattarocci, Paola Matarrese, Simona Anticoli, Claudia Abbruzzese, Stefania Catone, Rodolfo Sacco, Marco G. Paggi, Anna Ruggieri

**Affiliations:** Experimental Oncology, “Regina Elena” National Cancer Institute, IRCCS, Via Elio Chianesi, 53, 00144 Rome, Italy; Department of Molecular Biology, University of Geneva, 1211 Geneva, Switzerland; Department of Therapeutic Research and Medicines Evaluation, Istituto Superiore di Sanità, 00161 Rome, Italy; National AIDS Center, Istituto Superiore di Sanità, 00161 Rome, Italy; Gastroenterology and Metabolic Diseases, Department of Gastroenterology, 56124 Pisa University Hospital, Pisa, Italy; Department of Veterinary Public Health & Food Safety, Istituto Superiore di Sanità, 00161 Rome, Italy

**Keywords:** Hepatitis C Virus, Hepatocellular carcinoma, HCV core protein, *RBL2*, pRb2/p130, Cell cycle, Viral oncogenesis, Cell transformation

## Abstract

**Background:**

Hepatitis C Virus (HCV) infection is associated with chronically evolving disease and development of hepatocellular carcinoma (HCC), albeit the mechanism of HCC induction by HCV is still controversial. The nucleocapsid (core) protein of HCV has been shown to be directly implicated in cellular transformation and immortalization, enhancing the effect of oncogenes and decreasing the one of tumor suppressor genes, as *RB1* and its protein product pRB. With the aim of identifying novel molecular mechanisms of hepatocyte transformation by HCV, we examined the effect of HCV core protein on the expression of the whole Retinoblastoma (RB) family of tumor and growth suppressor factors, i.e. pRb, p107 and pRb2/p130.

**Methods:**

We used a model system consisting of the HuH-7, HCV-free, human hepatocellular carcinoma cell line and of the HuH-7-CORE cells derived from the former and constitutively expressing the HCV core protein. We determined pRb, p107 and pRb2/p130 protein and mRNA amount of the respective genes *RB1*, *RBL1* and *RBL2*, *RBL2* promoter activity and methylation as well as DNA methyltransferase 1 (DNMT1) and 3b (DNMT3b) expression level. The effect of pRb2/p130 over-expression on the HCV core-expressing HuH-7-CORE cells was also evaluated.

**Results:**

We found that the HCV core protein expression down-regulated pRb2/p130 protein and mRNA levels in HuH-7-CORE cells by inducing promoter hyper-methylation with the concomitant up-regulation of DNMT1 and DNMT3b expression. When pRb2/p130 expression was artificially re-established in HuH-7-CORE cells, cell cycle analysis outlined an accumulation in the G0/G1 phase, as expected.

**Conclusions:**

HCV core appears indeed able to significantly down-regulate the expression and the function of two out of three RB family tumor and growth suppressor factors, i.e. pRb and pRb2/p130. The functional consequences at the level of cell cycle regulation, and possibly of more complex cell homeostatic processes, may represent a plausible molecular mechanism involved in liver transformation by HCV.

## Background

Hepatitis C Virus (HCV), a member of the Flaviviridae family, has a single-stranded, positive-sense RNA genome of approximately 9.6 kb in length, which encodes a large polyprotein precursor of about 3,000 amino acids. This precursor is subsequently cleaved, by a combination of host and viral proteases, into at least ten proteins, four structural proteins [core, envelope 1 (E1), envelope 2 (E2) and p7] and six non-structural proteins (NS2, NS3, NS4A, NS4B, NS5A, and NS5B) [1, 2]. Infection by the hepatotropic HCV is a leading cause of chronic liver disease, with more than 170 million chronically infected individuals worldwide [3]. Chronic HCV infection is associated with the development of chronic hepatitis, fibrosis and cirrhosis, and is a major risk factor for the development and progression to hepatocellular carcinoma (HCC) [4].

Despite the successful development of the HCV sub-genomic replicon and the establishment of the JFH1 infectious virus model, the mechanisms underlying HCV-mediated tumorigenesis are still not fully understood [5]. It is known that the HCC development associated with HCV infection is a slow progressing oncogenic process, probably requiring multiple subsequent steps of genetic and epigenetic alterations. Indeed, activation of cellular oncogenes, inactivation of tumor suppressor genes, and dysregulation of multiple signal transduction pathways have been reported as possible pro-oncogenic mechanisms of HCV associated tumor [6]. Actually, virus-encoded factors establish a set of complex interactions with various cellular proteins and are actively implicated in several cellular signal transduction pathways that affect cell survival, proliferation, migration and transformation, thus contributing to viral persistence and pathogenicity. [7]. Among the HCV proteins, the 21 kDa core protein has been shown to modulate cellular genes expression, being involved in apoptosis, signal transduction, ROS formation, lipid metabolism, transcriptional activation, transformation and immune modulation [8–13]. HCV core binds to host tumor suppressor proteins, such as p53, p73 and pRb, modulates the expression of the cyclin dependent inhibitor p21^Waf1/Cip1^ [14, 15], a major target of p53, and regulates the activities of cyclin/cyclin-dependent kinase complexes involved in cell-cycle control and tumor onset/progression [16]. Furthermore, HCV core protein may also influence the growth and proliferation of host cells through activation of signaling pathways such as Raf/MAPK, Wnt/β-catenin and TGF-β, all known to be activated in HCC [17]. Lately, several studies have reported the presence of HCV core mutant proteins in HCV-infected patients who developed HCC, although the functional relevance of these mutations on malignant transformation is still not clear [18].

Due to the apparent involvement of the *RB1* gene and its product, pRb, in HCC onset and/or progression [19, 20], as well as the interplay with HCV infection and HCV core expression [21, 22], we sought to analyze the effect of the sole HCV core protein expression on all the Retinoblastoma (RB) family of tumor and growth suppressor factors, i.e. pRb, p107 and pRb2/p130, the protein products of the *RB1*, *RBL1* and *RBL2* genes, respectively [23–26]. These proteins are defined “pocket proteins”, due to the high homology they share at the level of the so-called pocket region, a domain fundamental for the accomplishment of their cellular effects [23, 24]. The pocket region is also the preferred target of several small DNA virus oncoproteins in order to overcome the growth suppressive properties of these endogenous factors [27]. The function of all the RB family proteins is post-translationally regulated by a complex modulation of their phosphorylation status [28]. They share overlapping functions, but possess also unique traits, often associated with cell and tissue types [24, 29, 30]. In particular, pRb2/p130 plays an important role in G0 non-proliferating cells, where it is found in its under-phosphorylated form, the one able to sequester and block its main E2F partners (E2F4 and E2F5). When cells re-enter the cell cycle, pRb2/p130 becomes phosphorylated and the release of the E2F partners reactivates the transcription of the cell growth-related genes [31, 32]. pRb2/p130 expression results altered in a number of human cancers, such as lung cancer [33, 34], endometrial [35], oral squamous cell carcinomas [36] and leukemias/lymphomas [37]. Available evidences support that pRb2/p130 may play an essential role in regulating growth and differentiation also in liver epithelial cells, and its elevated expression in HCCs, a context in which pRb is frequently down-regulated [30], has been considered as a possible protective mechanism to limit their uncontrolled growth [38]. Consistent with this, pRb2/p130 over-expression in HepG2 HCC cell lines results in G0/G1 cell cycle arrest, growth inhibition in vitro and *in vivo*, while its down-regulation appears thus implicated in the progression of the disease [39].

All the above observations prompted us to investigate on the effects of the HCV core protein on the RB family genes mRNA and protein expression in human HCC cell lines, with the aim to estimate the involvement of the RB family factors in the HCV core protein oncogenic mechanisms associated with HCV-induced carcinogenesis. To this end, we set up a model system composed of the HuH-7 human hepatocellular carcinoma cell line, originally established from an HCV-negative liver cancer patient [40–42], and the HuH-7-CORE cell clones, obtained from the former and capable to stably express the HCV core protein. This model appeared suitable for the investigation of cell cycle-related molecular mechanisms associated with the expression of the HCV core protein and allowed us to assess the ability of this viral protein in down-modulating the RB family proteins as well as its involvement in some molecular underlying events.

## Methods

### Cells transfection and plasmid construction

Human HCC cells HuH-7 were cultured at 37 °C in a 5 % CO_2_ atmosphere in Dulbecco’s modified Eagle’s medium (DMEM) supplemented with 10 % fetal bovine serum, 100 U/ml penicillin and 100 μg/ml streptomycin and 1X minimum essential amino acids solution (MEM). Cell cultures were passed twice a week and culture medium was changed every other day. Stable cell lines, expressing the HCV core protein, HuH-7-CORE, were established by transfection with Lipofectamine 2000 (Invitrogen Life Technologies, Carlsbad, CA) of pcCAG39neo (expressing the core protein) plasmid (constructed by A. Ruggieri), followed by selection with 400 μg/ml G418 (Geneticin, Gibco Invitrogen). In order to avoid the effect of G418 selected individual clones, the experiments were performed with a pool of clones obtained after the selection in G418 and by trypsinization of multiple clones from one plate.

pcDNA3Rb2/p130 sense and antisense expression plasmids [43] were transfected into HuH-7 and HuH-7-CORE cell lines with Lipofectamine 2000. Briefly, before transfection with pcDNA3Rb2 and *RBL2* (pRb2/p130) promoter constructs (see below for plasmid description), HCV core stable transfectants were cultivated in the absence of G418 for at least two passages. Cells were plated 24 h before transfection and grown to 80 % confluence in serum-free medium; cells were then incubated with Optimem medium (Invitrogen Life Technologies) under standard conditions for the first 6 h after transfection; then the medium was changed to DMEM containing 10 % fetal calf serum without antibiotics for another 48–72 h, when they were harvested for cell cycle analysis (Methods below).

pGL2bRb2P construct containing the full length Rb2/p130 promoter was obtained from PBS SK vector (kindly provided by Prof. A. Giordano) by excision, HindIII digestion, of the/HindIII digestion, of the 2,34 k b fragment, corresponding to the RBL2 promoter region. The excised fragment was inserted and ligated into the cloning site of pGL2-Basic vector (Promega, Madison, WI) that was linearized by HindIII/SacI digestion before ligation. The resulting plasmid, pGL2bRb2P, contained the *RBL2* promoter region whose functional expression could be evaluated by the induced expression of Firefly Luciferase gene downstream the cloning site. pGL2bRb2P was transfected into HuH-7 and in HuH-7-CORE cell lines using Lipofectamine 2000 as above. phRL-TK vector expressing Renilla Luciferase was co-transfected as an internal control for normalization of luciferase values.

### Western blot analysis

HuH-7 and HuH-7-CORE cells were harvested and dissolved in RIPA buffer (Tris-HCl 50 mM, NaCl 150 mM, 1 % sodium desoxycholate, 1 % Triton X-100, 0.1 % SDS, pH 7.5) or in pRb2/p130 buffer as previously described [43] supplemented with 1X proteinase inhibitor cocktail (Sigma Aldrich, Milan, Italy). Cell lysates (25 μg) were loaded onto a 10 % SDS-PAGE and analyzed under reduced conditions. Electrophoretically separated proteins were transferred on a PVDF membrane and probed with specific monoclonal antibody for 1.5 h at 37 °C or overnight at 4 °C. After extensive washing with TBS-T (Tris-HCl 20 mM, NaCl 137 mM, 0.5 % Tween, pH 7.6), membranes were incubated with appropriate HRP-conjugated secondary antibody. Protein bands were revealed by chemiluminescent substrates (ECL, Amersham Biosciences, Sweden). Antibodies: anti-HCV core protein mouse monoclonal antibody (Anogen Yes Biotech Laboratories. Ltd, Mississauga, Ontario, Canada) A1/3D1clone; anti-pRb2/p130 clone (BD Transduction Laboratories, Franklin Lakes, NJ); anti-pRb H-125 clone (Santa-Cruz Biotechnology, Santa Cruz, CA)¸ anti-p107 C-18 clone (Santa-Cruz); secondary antibody anti-mouse-HRP (Amersham Biosciences, Piscataway, NJ).

### Gene expression analysis by qRT-PCR

For the real-time quantitative RT-PCR, total cellular RNA was extracted from the HuH-7 and HuH-7-CORE cells using the Epicentre Master Pure RNA Purification kit according to the manufacturer’s instructions (Epicentre Biotechnologies, Madison, WI). Total RNA was digested with DNase I and then subjected to reverse transcription using high capacity cDNA Reverse Transcription kit (Applied Biosystems, Branchburg, NJ). Real-time quantitative PCR was performed using SensiMix SYBR kit (Bioline, Taunton, MA). The cycling program comprises initial denaturation at 95 °C for 10 min, 40 cycles of denaturation at 95 °C for 15 sec, annealing at 59 °C for 15 sec, extension at 72 °C for 15 sec and final extension at 72 °C for 5 min. DNA amplification was performed using a 7500 Fast Real-time PCR system (Applied Biosystems).

The primer sequences were as follows:

- *RBL1* (p107) forward: 5’-AAGTGTGAGCCGGTTAC-3’
- *RBL1* (p107) reverse: 5’-AGGATTACGCACACAAGA-3’
- *RB1* (pRb) forward: 5’-GAGCTTGGTTAACTTGGG-3’
- *RB1* (pRb) reverse: 5’-CAGATTCCCCACAGTTCC-3’
- *RBL2* (pRb2/p130) forward: 5’-GGGTGACTGAAGTTCGTGCT-3’
- *RBL2* (pRb2/p130) reverse: 5’-TGTGGTTGGAGATGTTATGCTC-3’

mRNA levels of all three RB family members were normalized using TATA-box binding protein (TBP). mRNA and relative expression profiles were generated using the comparative Ct method (ΔΔCT) [44, 45].

### Luciferase reporter assay

Dual luciferase Reporter Assay System (Promega) kit was used to measure the reporter luciferase activity induced by the expression of pRb2/p130 promoter in core expressing cell lines and in the control HuH-7, according to the protocol suggested by the manufacturer’s manual. Briefly, 5 × 10^4^ cells/well were plated in a 24-well plate, 24 h before transfection with 0.6 μg of pGL2bRb2P along with 70 ng/well of Renilla Luciferase expressing plasmid, as internal control. Forty-eight h after transfection, cells were lysed directly in wells with 1X PBL buffer and cell lysates were stored in aliquots at -80 °C or processed immediately for luciferase assay. For the assay, 20 μg of cell lysate were mixed with 100 μl of luciferase substrate, and light emission was measured with the LumiCount Luminometer (Perkin Elmer, Waltham, MA Life Sciences). The luciferase activity was normalized to protein concentration, determined by BCA method (Sigma-Aldrich). The value obtained was normalized to the Renilla-Luc activity measured in the corresponding cell extracts. Each experiment was repeated at least three times prepared in triplicate.

### DNA methylation analysis

Bisulfite treatment and recovery of genomic DNA samples were carried out with the EpiTect Bisulfite kit (Qiagen, Milan, Italy). Briefly, 2 μg of genomic DNA in 20 μl volume was used for each reaction and mixed with bisulfite mix (85 μl) and DNA protect buffer (35 μl). Bisulfite conversion was performed on a thermocycler as follows: 95 °C for 5 min, 60 °C for 25 min, 95 °C for 5 min, 60 °C for 85 min, 95 °C for 5 min, 60 °C for 175 min and 8 °C indefinitely. The bisulfite-treated DNA was recovered by EpiTect spin column by following the manufacturer’s instructions.

PCR, performed using pRb2/p130 primers that distinguish methylated (M) from unmethylated (U) sequences, was carried out in a 50 μl volume containing approximately 50 ng bisulfite-modified DNA, oligonucleotide primers (0.3 μM each) and 1x GoTaq Master Mix (Promega). DNA amplification was carried out with initial denaturing at 95 °C for 5 min, followed by 40 cycles of denaturing at 95 °C for 30 sec, annealing for 35 s at 55 °C and extension for 30 s at 72 °C. A final extension was added for 10 min at 72 °C after the last cycle. DNA products were electrophoresed on 2 % agarose gels and visualized by ethidium bromide staining.

Sequences to forward and reverse primers were as follows:

- *RBL2* (pRb2/p130)/U-For 5’-AACACAATACAAACAACAAACAAACAAACA-3’
- *RBL2* (pRb2/p130)/U-Rev 5’-GTTGTTTTAGGTTTTGGTTTGTGTTGTTTT-3’
- *RBL2* (pRb2/p130)/M-For 5’-GATACGAACGACGAACGAACGAACG-3’
- *RBL2* (pRb2/p130)/M-Rev 5’-TTTTAGGTTTCGGTTCGCGTCGTTTC-3’ [46].

The choice of two different experimental systems for luciferase and Methylation-Specific PCR (MSP) transcriptional assays is due to their specific technical features and responses.

### DNMT1 and DNMT3b mRNA expression analysis

Real-Time quantitative PCR assessment of DNMT1 and DNMT3b mRNA expression in HuH-7 and HuH-7-CORE cell lines was performed using the ABI PRISM 7500 Sequence Detection System (Applied Biosystems). 800 ng of total RNA were in vitro reverse-transcribed and 100 ng of cDNA was subjected to Real-Time quantitative PCR, performed in triplicate using TaqMan chemistry with primers and probe sets from the Assay-on-Demand list (Hs00154749_m1 and Hs00171876_m1 - Life Technologies). 18S RNA co-amplification was used as endogenous control reference.

### Cell cycle analysis

Cell cycle progression analysis of HuH-7 and HuH-7-CORE cell lines transiently expressing pRb2 and their relative controls was performed by flow cytometry, as previously described [47]. Cells were fixed and permeabilized with ice-cold ethanol for 30 min and then washed twice in PBS. DNA staining was performed by incubating cells at 37 °C in PBS containing 40 μg/ml propidium iodide and 0.4 mg/ml DNase-free RNase (type 1-A). Samples were analyzed by collecting FL2 red fluorescence in a linear scale at above 620 nm. The percentage of cells in the different phases of the cell cycle was determined by ModFIT software analysis. Apoptotic cells and debris were excluded by these analyses. Each experiment was repeated three times with duplicate samples.

### Statistical analysis

Results are expressed as a mean ± Standard Deviation (S.D.). Differences between groups were analyzed using the Student’s two-tailed *t* test (GraphPad Prism v5). Difference was considered to be significant if *P* < 0.05.

## Results

### HCV core expression and cellular localization in a human HCC cell line

**Fig. 1:**
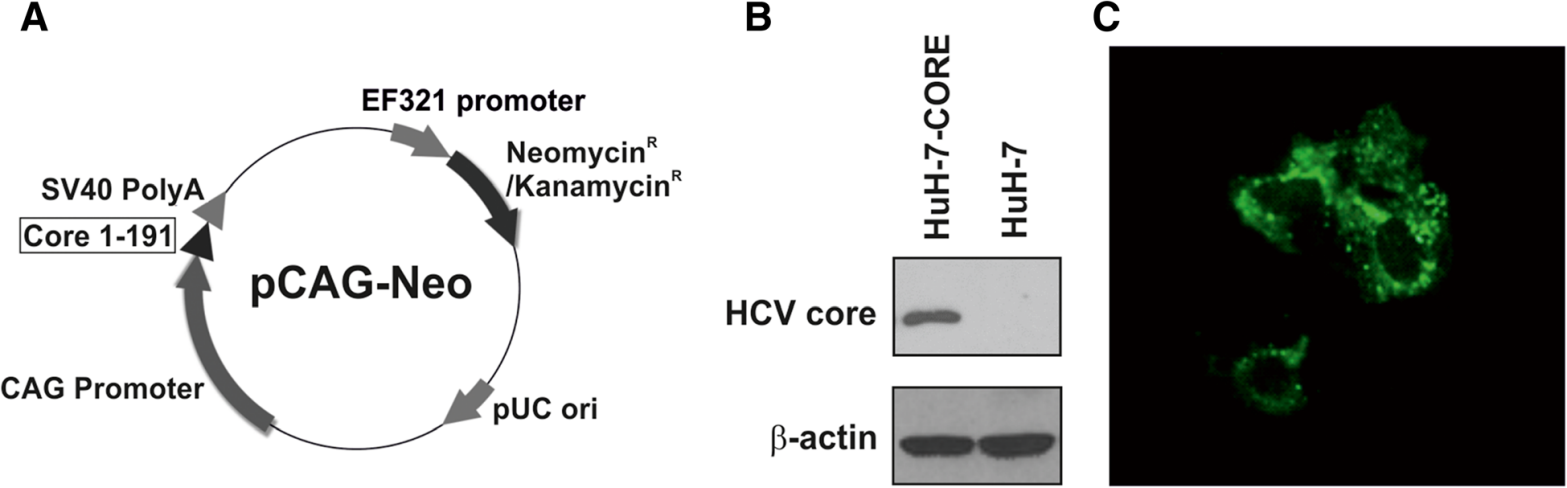
HCV core protein expression in HuH-7 stable transfectants. **a** Map of pcCAG39EFneo, the mammalian expression vector for cloning and expressing HCV core protein fragment (nt 1-191). **b** Western Blot detection of the HCV core protein constitutively expressed in HuH-7-CORE cell lysates. β-actin detection in the same blot is shown as loading control. **c** Immunofluorescence of the HCV core protein expression in HuH-7-CORE cell lines showing its peri-nuclear localization

To investigate the interplay between the HCV core protein and the cellular factors involved in cell growth and replication, we engineered the HCV-negative HuH-7 human HCC cell line in order to constitutively express high levels of the HCV core protein from genotype 1b, by transfection with the plasmid pcCAG39neo (Fig. 1, panel a). Transfected cell clones stably expressing the HCV core protein were selected in G418 and named HuH-7-CORE. The expression of the HCV core protein was assessed by Western blot (Fig. 1, panel b). HCV core appeared mainly localized in the cytoplasm, displaying a punctate distribution in the peri-nuclear area (Fig. 1, panel c).

### Modulation of the RB family proteins expression by the HCV core protein

HCV core protein is known to down-modulate pRb expression, the product of the *RB1* gene, in Rat-1 immortalized embryo fibroblasts [21] as well as its activity in other human liver and non-liver cell systems [22, 48].

**Fig. 2:**
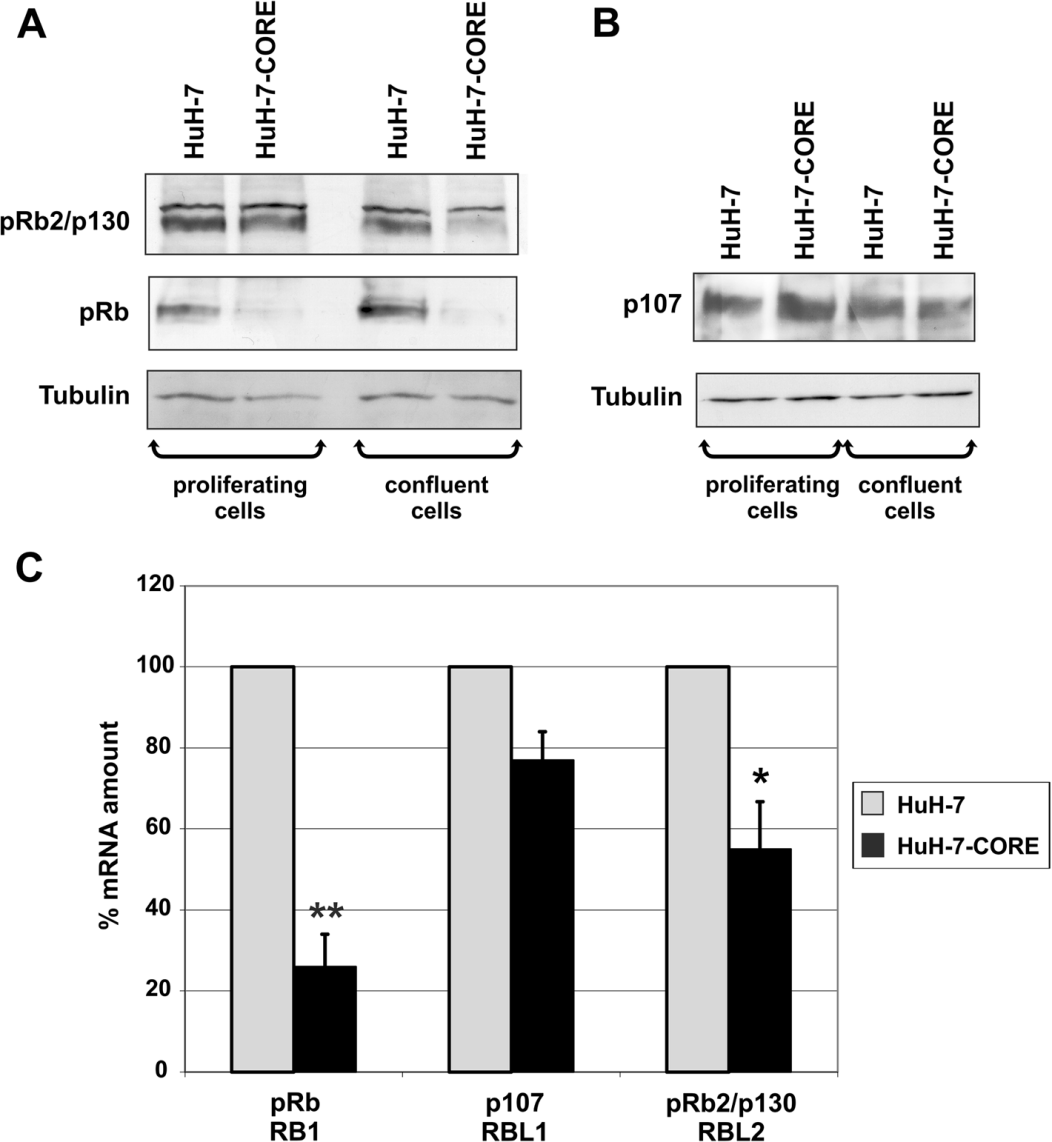
RB family proteins and mRNA modulation in HuH-7 parental and HuH-7-CORE cell lines. **a** and **b** Western blot analysis of pRb, pRb2/p130 and p107 proteins in parental HuH-7 and in core expressing HuH-7-CORE cell lines, showing a marked down-modulation of pRb and the phosphorylated form of pRb2/p130 in the HuH-7-CORE cell line. **c** Quantitative PCR determination of mRNA levels relative to the three RB family gene products, as indicated, in the parental HuH-7 cell line (grey bars, which refer to a 100 % relative amount) and in the HuH-7-CORE cells (black bars) constitutively expressing HCV core protein. Statistical significance: *P = <0.05; **P = <0.01

In order to examine the effect of the HCV core protein on the expression of all the RB family proteins, we determined by Western blot pRb, p107 and pRb2/p130 protein expression in both HuH-7 (parental) and HuH-7-CORE (HCV core-expressing) cells (Fig. 2). Cells were assayed during the logarithmic growth phase and after that confluence was reached in the culture dish. Consistent with literature data [21], HCV core strongly inhibited pRb expression in HuH-7-CORE cells (Fig. 2, panel a). Also pRb2/p130 appeared down-regulated in HCV core-expressing cells and, interestingly, such effect regarded essentially the under-phosphorylated, fast-migrating, form of the protein, the one able to exert growth suppressive properties [24, 49]. In the case of pRb2/p130, the effect resulted by far more evident when cells reached the confluence (Fig. 2, panel a). Conversely, under the same conditions, p107 expression appeared definitely less affected by the expression of the HCV core protein (Fig. 2, panel b). α tubulin determination was employed in both panels a and b as a loading control. p107 was always detected using a replica gel, due to its apparent molecular mass, and consequent gel migration, that partially overlapped with the one of pRb and pRb2/p130.

We thus determined by quantitative RT-PCR the mRNA levels related to the three RB family genes, *RB1*, *RBL1* and *RBL2*. In HuH-7-CORE cells, when compared with HuH-7 cells, results showed a significant reduction in mRNA levels for *RB1* and *RBL2*, while for *RBL1* the reduction appeared less evident (Fig. 2, panel c). Such mRNA expression results were quite consistent with the determination of the respective protein levels (Fig. 2, panels a and b).

### HCV core modulates RBL2 promoter activity

**Fig. 3:**
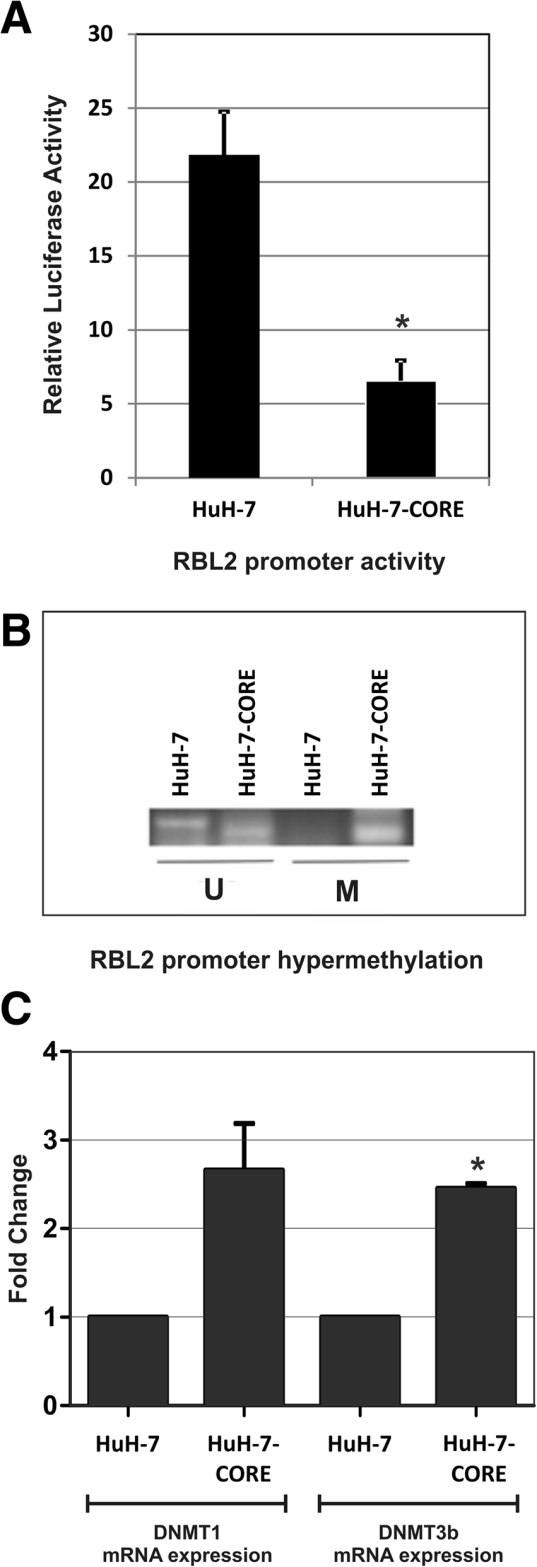
Evaluation of the *RBL2* promoter activity, its methylation status and DNMT1 and DNMT3b mRNA expression in HuH-7 parental and HuH-7-CORE cell lines. **a** The pGL2bRb2 construct, containing the full-length promoter region of *RBL2* linked to a luciferase reporter gene, was constructed and then transfected into HuH-7 and HuH-7-CORE cell lines. Measure of Luciferase activity (Luc/Renilla ratio) showed a more than three-fold down-modulation of *RBL2* promoter activity in HuH-7-CORE cells. The values plotted are the mean of three independent transfections, for each of which two different aliquots have been analyzed for Luciferase quantitation. Statistical significance: *P = <0.05. **b** Methylation-specific PCR (MSP) analysis covering the region abundant in CpG sequences was carried out on genomic DNA from HuH-7 and HuH-7-CORE cells. U = Unmethylated; M = Methylated. **c** qPCR determination of DNMT1 and DNMT3b mRNA expression shows its up-regulation in HuH-7-CORE cells with respect to the parental HuH-7 cell line, whose value has been normalized to 1. The results shown are the mean of two independent experiments performed in triplicate. Statistical significance: *P = <0.05

In search for the molecular mechanisms underlying pRb2/p130 down-regulation in HuH-7-CORE cells, we considered that HCV core is implicated, via promoter hypermethylation, in the negative modulation of several cellular genes [50–54]. Therefore, we sought to determine whether the decreased level of *RBL2* mRNA and protein found in the HuH-7-CORE cells could be related to a negative modulation of the *RBL2* promoter activity. We transfected the construct pGL2bRb2, containing the full length *RBL2* promoter region [43], linked to a luciferase reporter gene, into both HuH-7 and HuH-7-CORE cells. Evaluation of the promoter activity indicated that *RBL2* promoter was significantly down-regulated in the HuH-7-CORE cells when compared with the parental HuH-7 cells (Fig. 3, panel a), with more than three-fold decrease of the relative luciferase activity ratio (7.52 ± 1.34 vs. 23.90 ± 2.90, respectively with a statistical significance of *P* < 0.05).

### RBL2 epigenetic silencing by CpG methylation

DNA methylation of tumor suppressor genes has been described as one of the major epigenetic alterations in HCC [55] and such epigenetic modulations appear now also crucial for non-coding RNAs [56]. Considerably high incidence of promoter methylation in genes, like SOCS1 [57], GSTP1 [58, 59], APC [60], and CDKN2A (p16) [54] has been observed in HCV-positive HCCs [61, 62]. In addition, the HCV core protein appears able to up-regulate the levels of DNMT1 and DNMT3b and to induce promoter hypermethylation of tumor suppressor genes like E-cadherin and p16, resulting in down-regulation of their expression [51, 52, 54]. Thus, we sought to determine whether down-regulation of *RBL2* gene transcription in HCV core-expressing human HCC cell lines could be associated with 5’ CpG islands hypermethylation. MSP analysis covering the region abundant in CpG sequences (Fig. 3, panel b) indicated a significant *RBL2* promoter hypermethylation in HuH-7-CORE cells when compared with the parental HuH-7 cells, strongly suggesting the involvement of promoter hypermethylation as a cause of *RBL2* down-regulation in HuH-7-CORE cells.

To further determine the molecular mechanisms through which HCV core affected *RBL2* promoter methylation, we examined the mRNA expression of DNMT1 and DNMT3b that have been reported to positively influence HCV replication in HuH-7 cell line [63, 64]. Consistent with these previously reported results, we found an up-regulation of both DNMT1 and DNMT3b mRNA level in HuH-7-CORE cells, when compared with the parental HuH-7 cells (Fig. 3, panel c). In spite of an evident increase of transcription for both DNMT1 and DNMT3b, statistical significance was reached for DNMT3b expression only.

### Forced pRb2/p130 expression in HuH-7-CORE cells

The RB pocket proteins are the main controllers of the G1/S checkpoint in the cell cycle [24]. Consequently, their down-regulation by HCV core protein facilitates the transition of the cells toward the S phase. In order to estimate the specific role of the HCV core protein on the pRb2/p130 function, we over-expressed pRb2/p130 in the HuH-7-CORE cells through transfection of the pcDNA3RB2/p130 construct. After cytofluorimetric cell cycle analysis, we found that pRb2/p130 over-expression induced a slight, but statistically significant, increase in the percentage of the cells in G1, when compared with the mock-transfected HuH-7-CORE cells (Table 1). Concomitantly, an average decrease in the percentage of the S phase cells was detected. Analysis of cell cycle in HuH-7-CORE cells transfected with the *RBL2* antisense construct was included as a control. The amount of rescue elicited by pRb2/p130 over-expression in HuH-7-CORE cells can thus represent the functional role of the viral protein as a pRb2/p130 antagonist in the G1/S checkpoint dysregulation.

**Table 1:** HuH-7-CORE cells: cell cycle distribution

	Go/G1 (%)	S (%)	G2/M (%)
HuH-7-CORE (mock)	67 ± 5.8	24.5 ± 5.7	8.7 ± 2.2
HuH-7-CORE (pRb2/p130)	70 ± 7.0	21 ± 6.0	8.7 ± 0.9
HuH-7-CORE (pRb2/p130 as)	66.7 ± 5.6	24.6 ± 4.9	8.67 ± 0.69

Effect of pRb2/p130 over-expression on cell cycle distribution in HuH-7-CORE cells. HuH-7-CORE cells were transfected with a mock construct or with the pRb2/p130 construct. pRb2/p130 antisense construct (pRb2/p130 as) was also included as an additional control

## Discussion

HCC development in patients with chronic HCV infection is considered a multifactorial process, in which chronic liver inflammation (cirrhosis) and hepatocellular injury play an important role. However, HCC can still develop in a small proportion of non-cirrhotic patients with chronic hepatitis C infection, suggesting a direct involvement of HCV in hepatocarcinogenesis.

HCV-infected cells are forced by complex viral strategies to increase their survival ability and actively replicate, and HCV proteins are functional effectors evolved to obtain such goals. The present work enforces the notion that HCV core protein inhibits the G1/S transition of the cell cycle, demonstrates that pRb is not the only viral target to perturb this checkpoint and that a considerable role is played by targeting the sister protein pRb2/p130. Here we traced the flow of the events responsible for pRb2/p130 down-regulation by HCV core, going up to *RBL2* promoter hyper-methylation, a pretty common mechanism elicited by this viral protein. Such a loss in cell cycle control at the G1/S boundary exposes infected hepatocytes to repeated unscheduled mitoses that, combined with a reduction of the apoptotic processes, increase exponentially the risk of mitotic errors, as polyploidy, and ultimately neoplastic transformation [65]. Indeed, HCV is a cancer-associated RNA virus that functionally retraces some molecular mechanisms usually considered peculiar of the small DNA viruses [66], i.e. disruption of cell cycle checkpoints, down-regulation of key oncosuppressor genes, as pRb and p53, impairment of genomic integrity and mitotic machinery [67]. All these phenomena obviously concur in raising and maintaining a transformed cell clone. It is worth of note, in this context, that HCV core protein is constantly expressed in hepatocytes during HCV chronic infection and circulating anti-core antibodies are detectable in chronically infected patients.

## Conclusions

The observations we describe in the present work represent another step toward the comprehension of the molecular mechanisms of HCV-induced liver carcinogenesis. The RB family proteins are key regulators in pivotal cellular processes, e.g. cell cycle, apoptosis, genomic stability, senescence [23, 68] and the impairment of pRb functions by the HCV core protein has been widely described. Now we demonstrated that the pRb cognate protein pRb2/p130, which shares several functions with pRb, but shows also peculiar features [24], is likewise down regulated by HCV core and that this phenomenon is due, at least partially, to an increase in DNMT1 and DNMT3b activity, with consequent *RBL2* promoter hypermethylation and pRb2/p130 protein down-regulation. Now, another negative cell cycle regulator appears strongly impaired in its function, thus strengthening the possibility of a neoplastic drift.

Being HCV core a key factor in sustaining HCV infection and subsequent host cell transformation, a better knowledge at the molecular level of the effects of this viral protein on the cell machinery can help in combating HCV infection and HCV-related hepatocarcinoma as well.

## Abbreviations

DMEM: Dulbecco’s modified Eagle’s Medium
DNMT1: DNA methyltransferase 1
DNMT3b: DNA methyltransferase 3b
HCC: hepatocellular carcinoma
HCV: Hepatitis C Virus
MSP: Methylation-Specific PCR
PVDF: polyvinylidene difluoride
RB: retinoblastoma
ROS: reactive oxygen species
TBS: tris-buffered saline
